# High rate capability by sulfur-doping into LiFePO_4_ matrix†

**DOI:** 10.1039/c7ra12740e

**Published:** 2018-02-06

**Authors:** K. Okada, I. Kimura, K. Machida

**Affiliations:** Division of Applied Chemistry, Graduate School of Engineering, Osaka University Suita Osaka 565-0871 Japan machida@chem.eng.osaka-u.ac.jp; Department of Materials Science, Graduate School of Engineering, Osaka Prefecture University Sakai Osaka 599-8531 Japan

## Abstract

Enhanced electrochemical performance of LiFePO_4_ for Li-ion batteries has been anticipated by anion doping at the O-site rather than cation doping at the Fe-site. We report on the electrochemical performance of S-doped LiFePO_4_ nanoparticles synthesized by a solvothermal method using thioacetamide as a sulfur source. S-doping into the LiFePO_4_ matrix expands the lattice due to the larger ionic radius of S^2−^ than that of O^2−^. The lattice parameters *a* and *b* increase by around 0.2% with sulfur content, while that of *c* remains almost unchanged with only 0.03% increase. The S-doping also contributes to the suppression of antisite defects (Fe occupying Li sites), which facilitates the easy migration of Li in the diffusion channels without blockage. Owing to these effects of S-doping, the S-doped LiFePO_4_ nanoparticles show enhanced electrochemical properties with a high discharge capacity of ∼113 mA h g^−1^ even at a high rate of 10C.

## Abstract 2

S-doped LiFePO_4_ nanoparticles with improved electrochemical properties have been synthesized by a single step solvothermal method.

## Graphical abstract

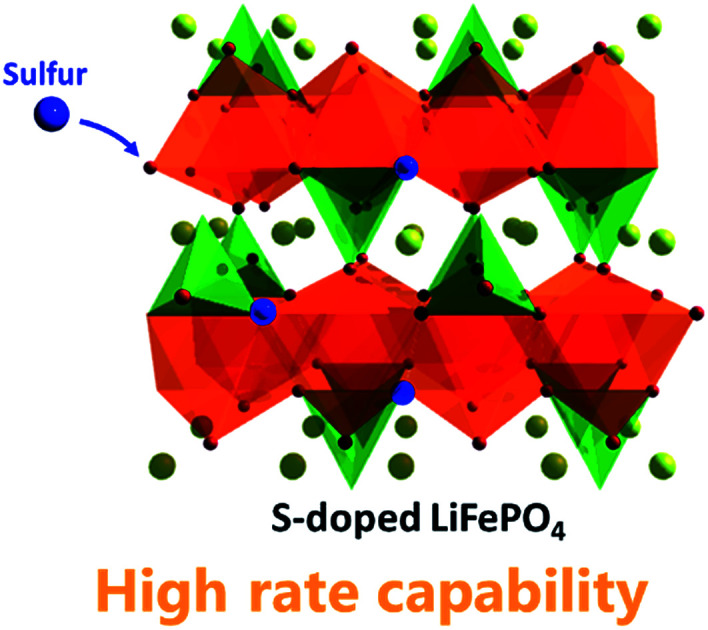

## Introduction

Rechargeable lithium-ion batteries (LIBs) are widely used for portable electronics and are also considered as promising energy storage devices for electric vehicles due to their long cycle life and high energy density.^1,2^ Ensuring safety of the devices and the use of environmentally-friend and cheap materials are important requirements to deal with further use of LIBs in the near future. Also, the current requirement for LIBs is to achieve both high charging/discharging rates and high capacity.^3^ Development of cathode materials plays an important role in satisfying these requirements because anode materials (mainly carbon materials) already exhibit high capacity at high charging/discharging rates.^4,5^

Olivine-structured LiFePO_4_ is considered as one of the most promising cathode materials for LIBs due to its high thermal stability, good cycling property, high energy density, theoretical capacity, and environmental friendliness as well as low cost.^6,7^ Although the theoretical capacity (170 mA h g^−1^) of LiFePO_4_ is a bit lower than that of conventional cathode materials (*e.g.*, LiCoO_2_: 274 mA h g^−1^), its benefits such as low toxicity, high safety, low-cost and good cycling property will make LiFePO_4_ replaced to conventional cathode materials as now being produced commercially.^8,9^ However, its poor electronic conductivity (10^−8^ to 10^−10^ S cm^−1^) and low lithium ion diffusion coefficient (10^−10^ to 10^−16^ cm^2^ s^−1^) lead to a decreased capacity at a high charging/discharging rate, which has inhibited the use in high-rate applications.^10–13^ Many efforts have been made to solve the problems by mainly three strategies: (1) carbon coating,^14,15^ (2) reducing particle size,^16,17^ (3) doping with certain element.^18–20^ Excellent electrochemical properties have been reported by combining these three strategies. The carbon coating can increase the surface electronic conductivity, leading to an improvement of rate performance and cycling life. The method of carbon coating has been developed and established already as a thermal treatment of the mixture of LiFePO_4_ and carbon sources under inert gas. Reducing particle size can shorten the Li^+^ diffusion pathway length, that is an effective way to improve rate capability. Hydro/solvo-thermal synthesis approaches have been applied to obtain the LiFePO_4_ nanoparticles. As for the doping strategy, doping proper ions into the LiFePO_4_ can increase the electronic and ionic conductivity of the LiFePO_4_ and also expand lithium ions diffusion channels in the structure, leading to a significant improvement of the rate capability and cyclic performance. Various elements such as La, Cu, Y, V, Mn, Mg, Bi, Co, Pt, Pd, Ni, Zn, Mo and Cr have been previously used as dopant elements in cation-site (Fe-site) of LiFePO_4_. Certain cation doping elements like Ni and Zn can remarkably enhance the electrochemical performance of LiFePO_4_.^21–24^ There are much less reports on anion doping at O-site for LiFePO_4_. However, the O-site doping is expected to significantly enhance the conductivity of LiFePO_4_ rather than Fe-site doping as indicated by first-principle calculations.^25,26^ Indeed, significantly enhanced high rate capability and cycling performance have been reported for anion-doped (such as F and Cl) LiFePO_4_.^27–29^ Among considerable anion species, sulfur doping would be efficient to improve the electrochemical performance (rate capability and cyclic performance) because easy Li^+^ intercalation and extraction are expected by an expansion of lattice arising from larger anion doping and the lower bond dissociation energy of Li–S (312.5 kJ mol^−1^) than that of Li–O (340.5 kJ mol^−1^).^30^ Sulfur-doping has been only reported by Park *et al.* where S-doped LiFePO_4_ nanoparticles exhibit enhanced electrochemical properties compared to non-doped and N-doped LiFePO_4_.^31^ However, only surface substitution of LiFePO_4_ nanoparticles was achieved by exposing solvothermally-synthesized LiFePO_4_ nanoparticles to sulfur vapor at high temperature. The further enhancement of electrochemical properties can be expected for the sulfur doping into the LiFePO_4_ matrix.

Herein, we report on the synthesis of S-doped LiFePO_4_ nanoparticles by a single-step solution approach and its improved electrochemical performance compared to non-doped LiFePO_4_. Thioacetamide was used as a sulfur source, which was the conventional way for the synthesis of S-doped TiO_2_ and metal sulfide such as CdS, ZnS and Bi_2_S_3_ by the solution approach.^32–35^ The successful S-doping into LiFePO_4_ matrix was confirmed by X-ray diffraction (XRD), energy dispersive X-ray spectrometry (EDS) analysis and X-ray photoelectron spectrometry (XPS). The formation of LiFePO_4_ nanoparticles with ∼100 nm in diameter was observed by scanning electron microscopy (SEM). The S-doped LiFePO_4_ nanoparticles showed a high discharge capacity of ∼113 mA h g^−1^ even at a high rate of 10C.

## Experimental

### Preparation of non-doped and S-doped LiFePO_4_ nanoparticles

Non-doped and S-doped LiFePO_4_ nanoparticles were prepared by a solvothermal method. The non-doped LiFePO_4_ nanoparticles were prepared as following the literature.^36^ At first, 6 mmol of FeSO_4_·7H_2_O and 6 mmol of H_3_PO_4_ (85%) were completely dissolved in 20 mL of ethylene glycol. At same time, 16.2 mmol of LiOH·H_2_O was also dissolved in 15 mL of ethylene glycol. Then, the solution containing a Li source was added dropwise to the solution containing Fe and P sources. The final molar ratio of Li : Fe : P was 2.7 : 1 : 1. The reaction was carried out in an autoclave at 180 °C for 10 h. After the reaction, the resultant gray precipitate was washed with deionized water and ethanol for several times and then dried at 70 °C overnight under vacuum. S-doped LiFePO_4_ nanoparticles were prepared by adding thioacetamide with different amount (0.13, 0.40, 0.67, 1.33, 2.66 and 3.99 mmol) to the reaction solution containing Li, Fe and P sources before solvothermal treatment (the molar ratio; Li : Fe : P : S = 2.7 : 1 : 1 : 0.02, 0.07, 0.11, 0.22, 0.44 or 0.67). In the manuscript, the non-doped and S-doped LiFePO_4_ nanoparticles prepared with different amount of thioacetamide were denoted as LFP and LFP-S-*x* (where *x* = 0.02, 0.07, 0.11, 0.22, 0.44 or 0.67), respectively. For the electrochemical measurements, the dried samples with a certain amount of sucrose (10 wt%) were mixed in ethanol and calcined at 650 °C for 6 h under N_2_ atmosphere in order to obtain the carbon-coated samples.

### Characterization

The crystalline phases of samples were identified by X-ray diffraction (XRD; Rigaku, RINT-2200) using a Cu Kα radiation. The unit cell parameters were investigated by Rietveld refinement (using the RIETAN-FP program).^37^ Surface morphologies of samples were observed using a scanning electron microscope (SEM, Hitachi Corp., S-3000HXS); the energy dispersive X-ray analysis has been performed using 15 keV. X-ray photoelectron spectrometry (XPS) measurements were carried out using an electron spectrometer (Shimadzu Corp./Kratos, AXIS-165×) with an Al Kα X-ray source. The position of each peaks was collected by using C spectra. Fourier transform infrared spectroscopy (FTIR) measurements were carried out using a FTIR 4700 spectrometer (JASCO).

#### Electrochemical measurements

The electrochemical performances were measured by using an assembly cell (Hohsen, Japan) with lithium foil as counter and reference electrodes. For the preparation of working electrodes, at first, the slurries of carbon-coated non-doped and S-doped LiFePO_4_ were prepared by mixing active materials, acetylene black, and polyvinylidene difluoride (PVDF) binder (a mass ratio of 6.5 : 2.5 : 1) in *N*-methyl-2-pyrrolidone (NMP) solution. Then, the slurry was coated on an Al foil by using a coater. After being dried under vacuum at 90 °C for 10 h, working electrodes were punched and weighed. Then, the cells containing 1.0 mol L^−1^ LiPF_6_ in ethylene carbonate (EC)-diethyl carbonate (DEC) (1 : 1, v/v) as electrolyte were assembled in a glove box under a dry and high purity argon atmosphere. Charge/discharge tests were performed between 2.0 and 4.3 V by using a battery tester (Kikusui, PFX2011, Japan) at room temperature at around 20 °C.

## Results and discussion

**Fig. 1:**
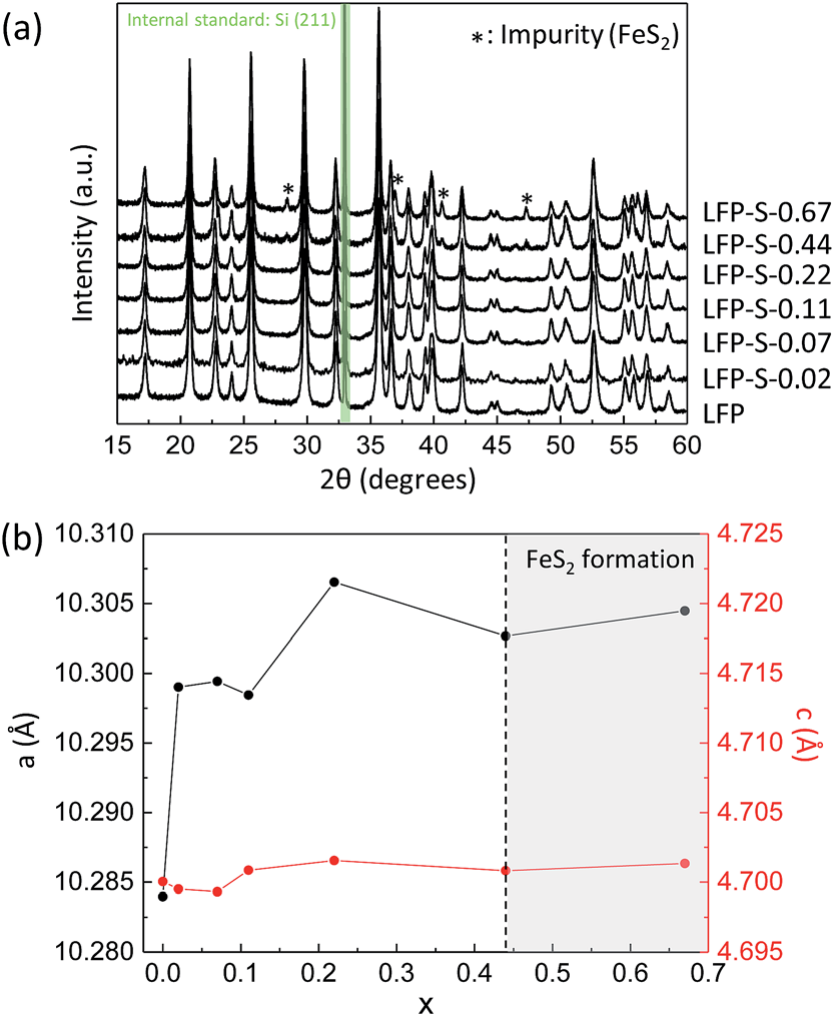
(a) XRD patterns of LFP and LFP-S-*x* (where *x* = 0.02, 0.07, 0.11, 0.22, 0.44 or 0.67) (silicon was used as a standard). (b) Changes in *a* and *c* lattice parameters of LFP-S-*x* (*x*: from 0 to 0.67).

Non-doped and S-doped LiFePO_4_ nanoparticles were prepared by a solvothermal method. The non-doped LiFePO_4_ nanoparticles were prepared from lithium, iron and phosphorus sources dissolved in ethylene glycol at 180 °C for 10 h as following the literature.^36^ For the sulfur doping, thioacetamide was added to the reaction solution. Hereafter, the non-doped and S-doped LiFePO_4_ nanoparticles prepared with different amount of thioacetamide were denoted as LFP and LFP-S-*x* (where *x* = 0.02, 0.07, 0.11, 0.22, 0.44 or 0.67), respectively. Fig. 1(a) shows XRD patterns of the obtained samples (where silicon was used as a standard). The peaks of LFP and LFP-S-*x* (*x* ≤ 0.22) were exclusively indexed to the LiFePO_4_ phase (JCPDS card number: 81-1173). While, the formation of FeS_2_ (JCPDS card number: 42-1340) was detected as an impurity for the samples prepared with an excess of thioacetamide (*x* ≥ 0.44). The unit cell parameters were investigated by Rietveld refinement (using the RIETAN-FP program).^37^ An increase of lattice parameters with sulfur doping amount was confirmed as shown in Fig. 1(b) and Table S1,† indicating the successful doping of sulfur in LiFePO_4_ matrix because the ionic radius of S^2−^ (1.84 Å) is larger than O^2−^ (1.40 Å).^38^ Interestingly, the lattice parameters of *a* and *b* were found to increase by around 0.2% with sulfur doping, while that of *c* remained almost unchanged as only 0.03% increase. Such large changes of *a* and *b* lattice parameters were similarly reported for the F-doping into LiFePO_4_.^39^ In the case of cation doping at Fe-site, the most cationic elements (except vanadium) are found to change the lattice parameters uniformly in literature.^40–45^ Thus, it should be noted that the anion doping at O-site can specially change the *a* and *b* lattice parameters and remain *c* lattice parameter unchanged, which is not observed for cation doping. That might be explained by the position of oxygen atoms in the LiFePO_4_ lattice. Oxygen atoms are located parallel to the *a* and *b* axes of LiFePO_4_ lattice while irregularly for the *c* axis.^46^ Thus, it can be considered that the sulfur doping at O-site simply expands the lattice along the both *a* and *b* axes and the stress of *c* axis induced by the sulfur doping are released toward *a* and *b* axes by distortion, which would induce the specific changes for *a* and *b* axes of LiFePO_4_ lattice. The expansion of LiFePO_4_ lattice by sulfur doping was maintained after carbon coating at 650 °C under inert atmosphere (Fig. S1†). A further increase in *a* and *b* lattice parameters was observed after the carbon coating process. The thermal treatment at 650 °C might make the crystal structure more stable by displacing the S atoms into favored positions and minimizing ion disorder.^47^ The expansion of LiFePO_4_ lattice by a sulfur doping would give us the benefit of high capacity at high charging/discharging rate because of easy Li^+^ intercalation and extraction by expanded lithium ions diffusion channels.

**Fig. 2:**
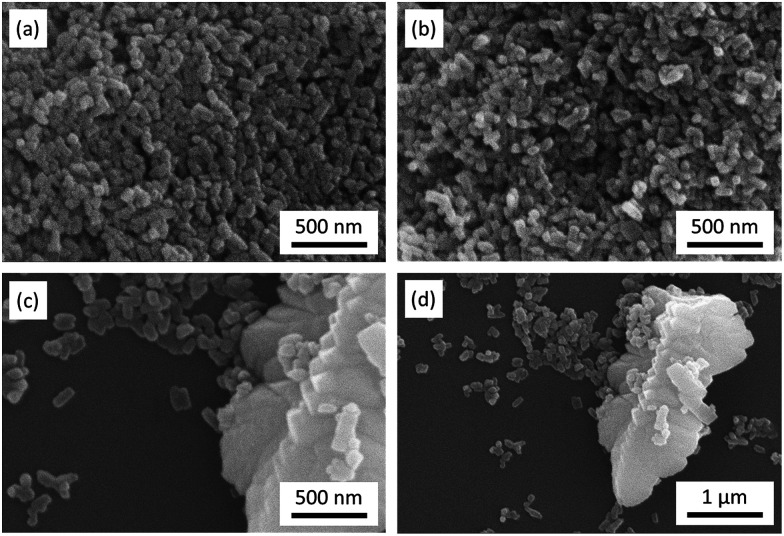
SEM images of LFP (a), LFP-S-0.22 (b) and LFP-S-0.67 (c and d).

**Fig. 3:**
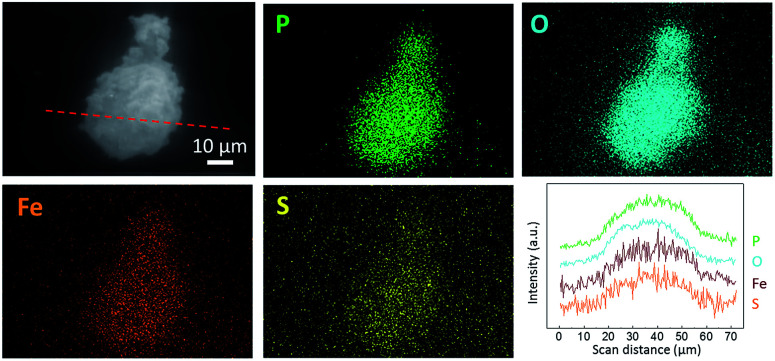
Secondary electron image (SEI), EDS mapping images and line scan profile of LFP-S-0.22 for P, O, Fe and S. The line scan profile was collected in the red dot line of the SEI image.

Fig. 2 shows the SEM images of LFP, LFP-S-0.22 and LFP-S-0.67. Uniform nanoparticles with ∼100 nm in diameter were observed for LFP (Fig. 2(a)). Similar nanoparticles formed in LFP-S-0.22 (Fig. 2(b)), indicating S-doping did not affect the morphology. In the LFP-S-0.67, larger and crystalline particles over 1 μm were observed with the presence of LiFePO_4_ nanoparticles (Fig. 2(c) and (d)). The large particles were concluded as an impurity FeS_2_ by the XRD result and EDS analysis (Fig. S2†). EDS mapping and analysis for S-doped LiFePO_4_ (LFP-S-0.22) confirmed that sulfur was uniformly dispersed as an atomic percent of ∼1.4% (Fig. 3 and S3†). Based on this result, the composition of LFP-S-0.22 was estimated as LiFePO_3.9_S_0.1_.

**Fig. 4:**
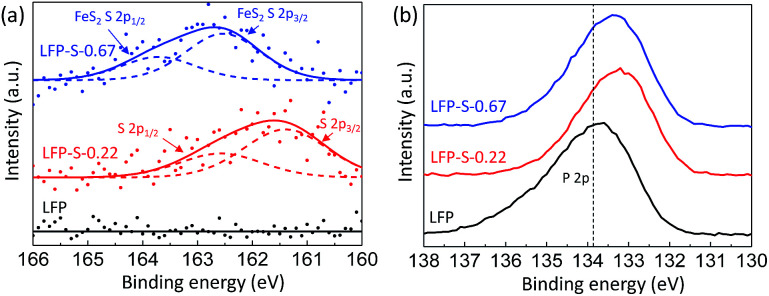
XPS spectra for the S 2p (a) and P 2p (b) electrons of LFP, LFP-S-0.22 and LFP-S-0.67.

The chemical states of sulfur, phosphorous and oxygen ions were investigated by XPS (Fig. 4). Fig. 4(a) shows the XPS spectra for the S 2p electrons of LFP, LFP-S-0.22 and LFP-S-0.67. A broad peak at around 160–163 eV was confirmed in the LFP-S-0.22 although no peaks was detected in LFP. The peak was fitted as the core levels of S 2p_1/2_ (at 162.4 eV) and S 2p_3/2_ (at 161.4 eV) separated by a spin–orbit splitting of 1.0 eV, demonstrating that the sulfur atoms are doped in the state of S^2−^.^48,49^ On the other hand, a broad peak at ∼1.0 eV higher than that of LFP-S-0.22 was detected in LFP-S-0.67. The peak at higher energy could be assigned to that of disulfide derived from FeS_2_.^50,51^ The presence of FeS_2_ in LFP-S-0.67 was consisted with the XRD results. The XPS spectra for the P 2p electrons of LFP, LFP-S-0.22 and LFP-S-0.67 are shown in Fig. 4(b). As shown in Fig. 4(b), the sulfur doping shifted the P 2p XPS peak to lower binding energy. Similar chemical shift of binding energy has been reported for the nitrogen doping at Li_3_PO_4_ and Na_3_PO_4_ where a decrease in the P 2p binding energy was explained by simple charged-shell models^52,53^ as reducing the average ionic charge on the phosphorus ions due to the replacement of P–O bonds with less ionic P–N bonds.^54,55^ The binding energy chemical shift by sulfur doping to LiFePO_4_ can be explained by the similar effect; reducing the average ionic charge on the phosphorus ions due to the replacement of P–O bonds with less ionic P–S bonds results in the lower P 2p binding energy. O 1s XPS peaks in LFP-S-0.22 also showed lower binding energy compared to LFP, which is similar to N-doped Li_3_PO_4_ and Na_3_PO_4_ (ref. 54) (Fig. S4†). These XPS results for the S 2p, P 2p and O 1s electrons clearly indicate the successful S^2−^ doping at O-site of LiFePO_4_ matrix.

**Fig. 5:**
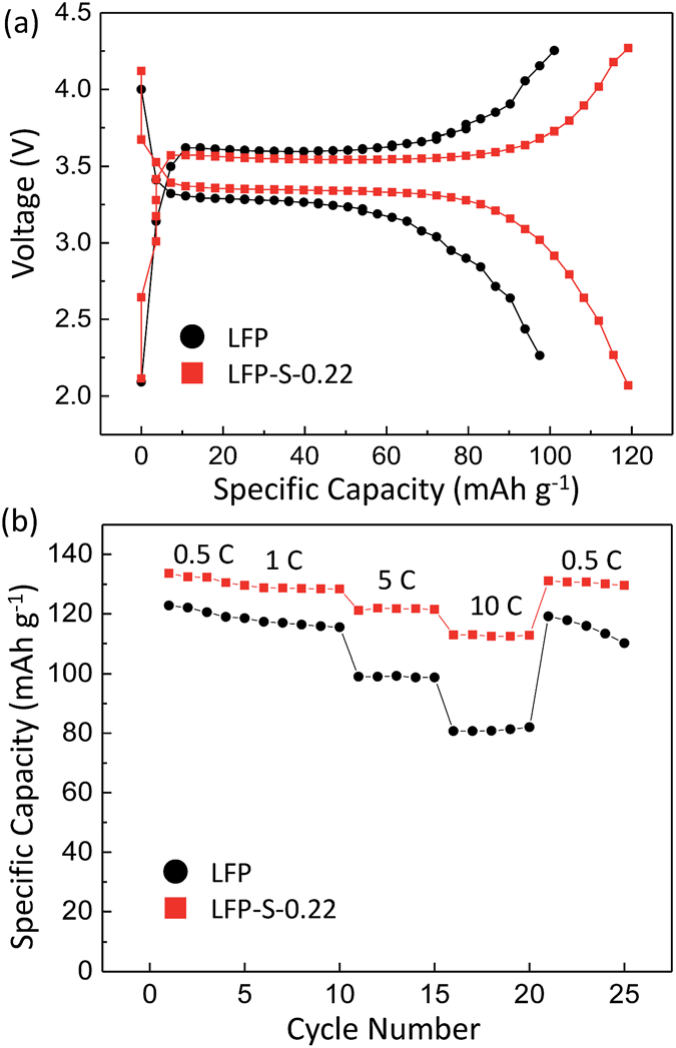
(a) Charge–discharge voltage curves of LFP and LFP-S-0.22 at 5C. (b) Rate performance of LFP and LFP-S-0.22.

**Fig. 6:**
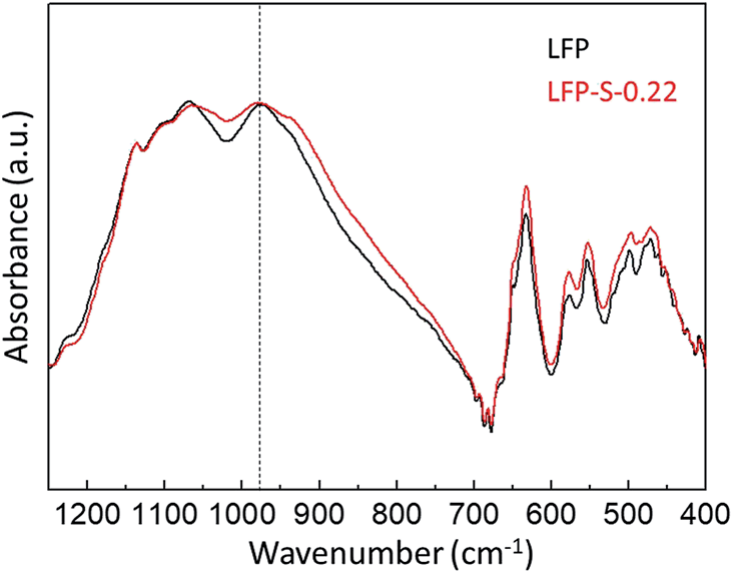
FTIR spectra of LFP and LFP-S-0.22.

Electrochemical measurements were carried out to test the electrical properties of LFP and LFP-S-0.22 after carbon coating by following a reported method.^56^Fig. 5(a) shows the charge/discharge curves of carbon-coated LFP and LFP-S-0.22 measured at 5C (850 mA g^−1^) between 2.0 and 4.3 V *vs.* Li^+^/Li. Both samples showed a charging and discharging plateau region at ∼3.4 V which is correspond to the redox reaction between FePO_4_ and LiFePO_4_.^57^ A smaller voltage difference between the charge and discharging plateaus was confirmed in LFP-S-0.22 compared to LFP, implying that LFP-S-0.22 possess the better electronic conductivity and higher reaction reversibility.^58^ The non-doped LFP presented a discharge capacity of 99.0 mA h g^−1^ at 5C. In contrast, S-doped LFP-S-0.22 led to a higher discharge capacity of 121.6 mA h g^−1^ at 5C. The discharge capacities of LFP and LFP-S-0.22 were investigated at different rates from 0.5C to 10C in order to examine the rate capabilities (Fig. 5(b)). The LFP-S-0.22 showed much higher discharge capacities (131.7, 128.5, 121.6 and 112.7 mA h g^−1^ at 0.5, 1, 5 and 10C, respectively) at each rates compared to LFP (120.6, 116.4, 99.0 and 81.1 mA h g^−1^ at 0.5, 1, 5 and 10C, respectively). In addition, the discharge capacity of LFP-S-0.22 varied much smaller from 128.5 to 112.7 mA h g^−1^ at rates of 1C to 10C, retaining 87.7% of discharge capacity. There was no difference between LFP and LFP-S-0.22 for coulombic efficiency at each C rate (Fig. S5†). These electrochemical investigations exhibited that sulfur doping into LiFePO_4_ effectively improved the rate capability. It has been considered that high rate capability can be achieved by an increase in electric conductivity of LiFePO_4_. The significantly enhanced high rate capability achieved in LFP-S-0.22 was considered due to an increase in electric conductivity by sulfur doping as expected by first-principle calculations.^31^ The differential capacity (d*Q*/d*V*) studies showed smaller peak voltage separation between the anodic and cathodic peaks in LFP-S-0.22 compared to LFP, indicating the smaller electrochemical polarization and better electrochemical kinetics of the S-doped LiFePO_4_ (Fig. S6†).^59,60^ Also, the LFP-S-0.22 exhibited higher internal electronic conductivity and lower resistance than LFP (Fig. S7†). In addition, the sulfur doping results in the suppression of 
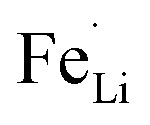
 antisite defects (Fe occupying Li sites) in LiFePO_4_ which is important as it facilitates the easy migration of Li in the diffusion channels without blockage.^61^ The antisite defect is considered to be present in the one-dimensional channel of LiFePO_4_ and effectively block the Li^+^ pathways,^62–64^ which reduces the capacity, rate capability and cycle life of a battery. The suppression of 
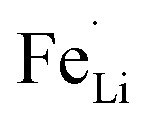
 antisite defects can be investigated by Fourier transform infrared (FTIR) spectra.^39,65^Fig. 6 shows the FTIR spectra of LFP and LFP-S-0.22. The Infrared absorption spectra in the range 800–1200 cm^−1^ and 400–700 cm^−1^ were assigned to the symmetric (*ν*_1_) and asymmetric (*ν*_3_) P–O stretching, symmetric (*ν*_2_) and asymmetric (*ν*_4_) O–P–O bending in (PO_4_)^3−^ tetrahedral, respectively.^66^ As shown in Fig. 6, the sulfur doping shifted a peak at ∼975 cm^−1^ to lower wavenumber, while there were no significant changes for the other peaks. This result is clearly consistent with the 
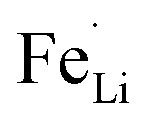
 antisite defects-suppressed LiFePO_4_, suggesting sulfur doping into the LiFePO_4_ matrix can also suppress the formation of 
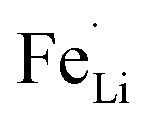
 antisite defects. In the LiFePO_4_ structure, lithium and iron ions are surrounded by six oxygen atoms and occupy the different octahedral interstitial sites; Li ions occupy edge-sharing sites (M1) and Fe ions occupy corner-sharing sites (M2). The 
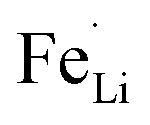
 antisite defects reportedly form by cation intermixing (cation-exchanging) between the M1 and M2 sites.^62^ Our results indicate that the sulfur doping at O-site makes the Fe ions preferentially located in the M2 sites. In the reaction media, the sulfur prefers to react with iron ions as our XRD investigation revealed the formation of FeS_2_ with excess of sulfur sources and also the preferential formation of Fe–S bonds was reported in the sulfur-modified LiFePO_4_ on the surface by theoretical and experimental investigations.^31^ Then, the formed Fe–S species react with the H_3_PO_4_ by replacing O to S in the reaction media, which results in the preferential location of Fe in M2 sites by corner-sharing oxygen or sulfur atoms with PO_4_ tetrahedral. Consequently, the occupation of Fe in the M1 sites (
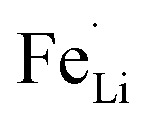
 antisite defects) would be suppressed by sulfur doping at O-site. The suppression of 
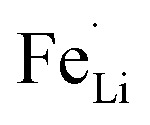
 antisite defects by S-doping would also contribute to improvement in the capacity and the rate capability compared to non-doped LiFePO_4_. It should be also mentioned that the discharge capacity of 112.7 mA h g^−1^ at 10C achieved in LFP-S-0.22 is much higher than that of sulfur-doped LiFePO_4_ only on the surface (86.4 mA h g^−1^ at 10C) reported in the literature.^31^ Thus, it can be concluded that anion doping into the LiFePO_4_ matrix is more important than anion doping only on the surface of LiFePO_4_ particle in order to enhance the electrochemical performance. The synthesis of cation-doped LiFePO_4_ has been reported by similar solvothermal approaches.^67,68^ Thus, our system would be simply applied to the cation-doping approach. The further enhancement of electrochemical performance is expected by multi-elements co-doped LiFePO_4_ with certain elements.

## Conclusions

In this work, we successfully prepared the S-doped LiFePO_4_ nanoparticles with ∼100 nm in diameter by a solvothermal method using thioacetamide as a sulfur source. The LiFePO_4_ lattice is expanded with increasing the sulfur amount, but the excess amount of sulfur results in the formation of FeS_2_ as an impurity. The lattice parameters of *a* and *b* increase by around 0.2% with the sulfur content, while that of *c* remains almost unchanged as only 0.03% increase. The sulfur doping at O-site also results in the suppression of 
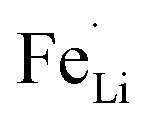
 antisite defects in lithium ions diffusion channels. Both the lattice expansion and the suppression of 
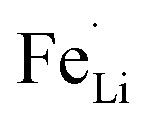
 antisite defects by S-doping contribute to the enhancement of electrochemical properties due to the easy migration of Li in the diffusion channels without blockage.

## Conflicts of interest

There are no conflicts to declare.

## Supplementary Material

RA-008-C7RA12740E-s001
