# Supplementary material for: High rate capability by sulfur-doping into LiFePO4 matrix
Source: RSC Adv. 2018 Feb 6;8(11):5848–53. doi: 10.1039/c7ra12740e (PMC9078183; doi:10.1039/c7ra12740e)
Supplement: RA-008-C7RA12740E-s001 [file RA-008-C7RA12740E-s001.pdf]

Electronic Supplementary Material (ESI) for RSC Advances.
This journal is © The Royal Society of Chemistry 2018

Supporting Information

High rate capability by sulfur-doping into $LiFePO_4$ matrix

Kenji Okada[ab], *Ikumi Kimura*[a], *Ken-ichi Machida*[a]*

[a] *Department of Applied Chemistry, Graduate School of Engineering, Osaka University, Suita, Osaka, 565-0871, Japan*
[b] *Department of Materials Science, Graduate School of Engineering, Osaka Prefecture University, Sakai, Osaka 599-8531, Japan*

Table S1. Calculated lattice parameters LFP and LFP-S-x (where x=0.02, 0.07, 0.11, 0.22, 0.44 and 0.67) by Rietveld refinement (using the RIETAN-FP program).

Sample name	*a* (Å)	*b* (Å)	*c* (Å)	V ($Å^3$)	R_{wp}
LFP	10.28398	5.98292	4.70004	289.185	4.972
LFP-S-0.02	10.29902	5.99173	4.69951	290.0019	5.866
LFP-S-0.07	10.29943	5.9921	4.69932	290.0196	5.478
LFP-S-0.11	10.29845	5.99102	4.70085	290.034	5.678
LFP-S-0.22	10.30653	5.9958	4.70155	290.5361	5.568
LFP-S-0.44	10.30267	5.99314	4.70082	290.2536	6.773
LFP-S-0.67	10.30447	5.99312	4.70134	290.3358	5.082

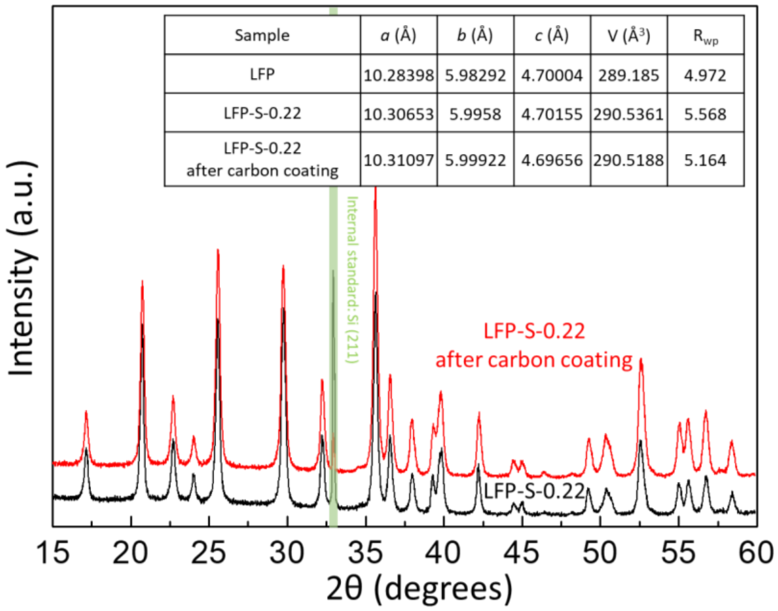

Figure S1. XRD patterns of LFP-S-0.22 and carbon-coated LFP-S-0.22.

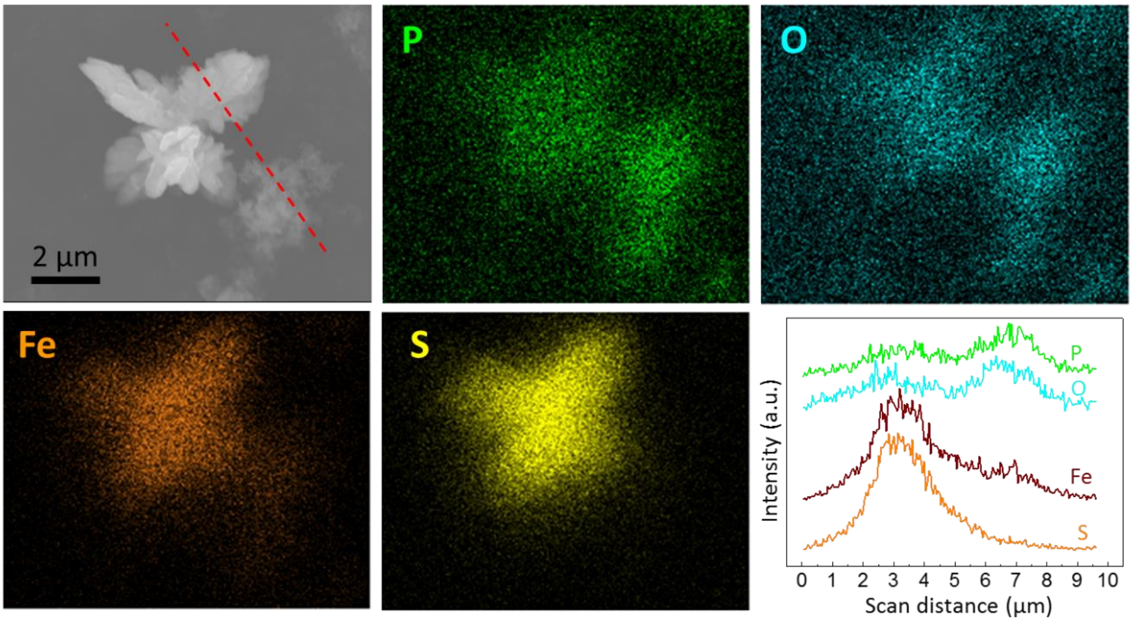

Figure S2. Secondary electron image (SEI), EDS mapping images and line scan profile of LFP-S-0.67 for P, S, Fe and S. The line scan profile was corrected in the red dot line of the SEI image. These clearly indicate that the large and crystalline particles over 1 μm observed in LFP-S-0.67 are consisted of Fe and S. Thus, the particles are concluded as an impurity FeS_2 taking into account of XRD results.

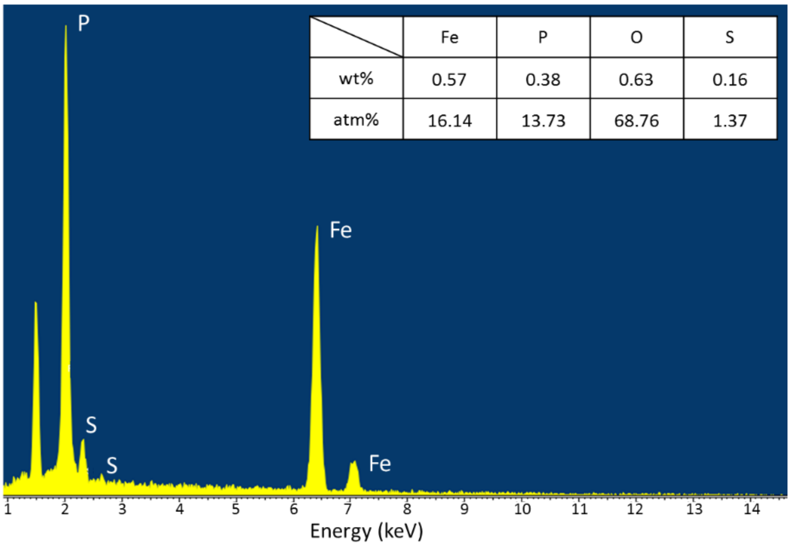

Figure S3. EDS spectrum from LFP-S-0.22.

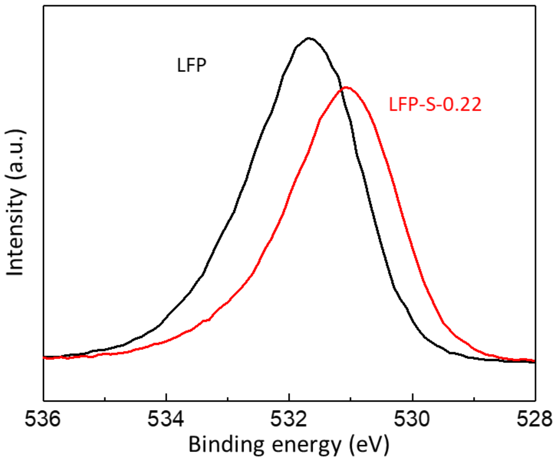

Figure S4. XPS spectra for the O 1s electron of LFP, LFP-S-0.22 and LFP-S-0.67.

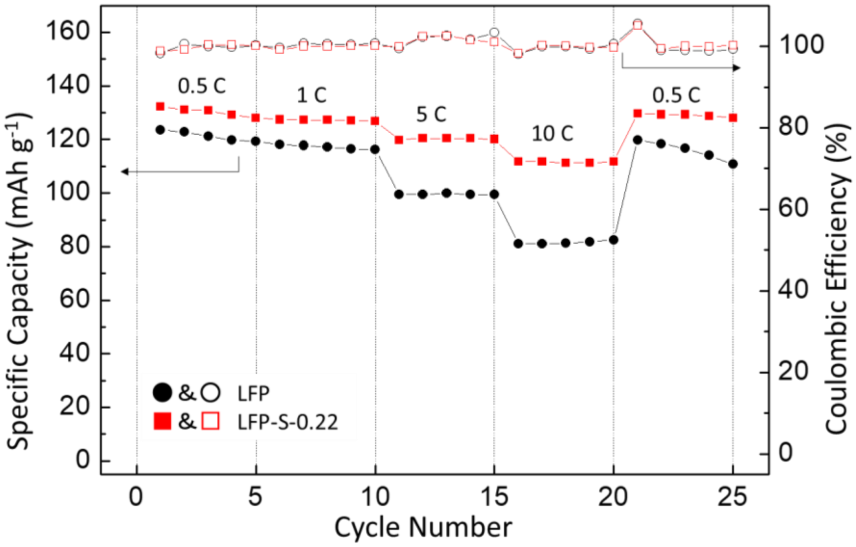

Figure S5. Rate performance and Columbic efficiency of LFP and LFP-S-0.22. Columbic efficiency at each C rates was calculated based on a report by Smith et. al. [A. J. Smith, J. C. Burns and J. R. Dahn, *Electrochem. Solid-State Lett.*, 2010, 13, A177].

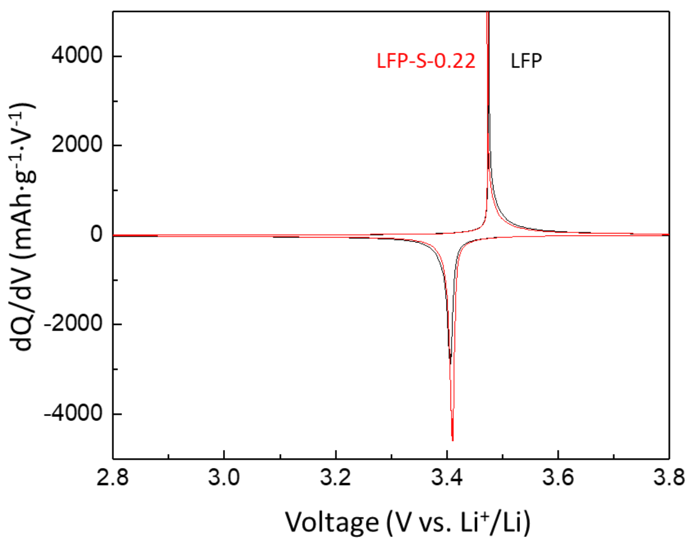

Figure S6. Differential capacity (*dQ/dV*) versus voltage profiles of LFP and LFP-S-0.22 electrodes at 1C. LFP showed cathodic peaks at 3.475 V and anodic peaks 3.405 V. LFP-S-0.22 showed cathodic peaks at 3.474 V and anodic peaks 3.410 V. The LFP-S-0.22 has smaller peak voltage separation than LFP, indicating the smaller electrochemical polarization and better electrochemical kinetics of the S-doped $LiFePO_4$

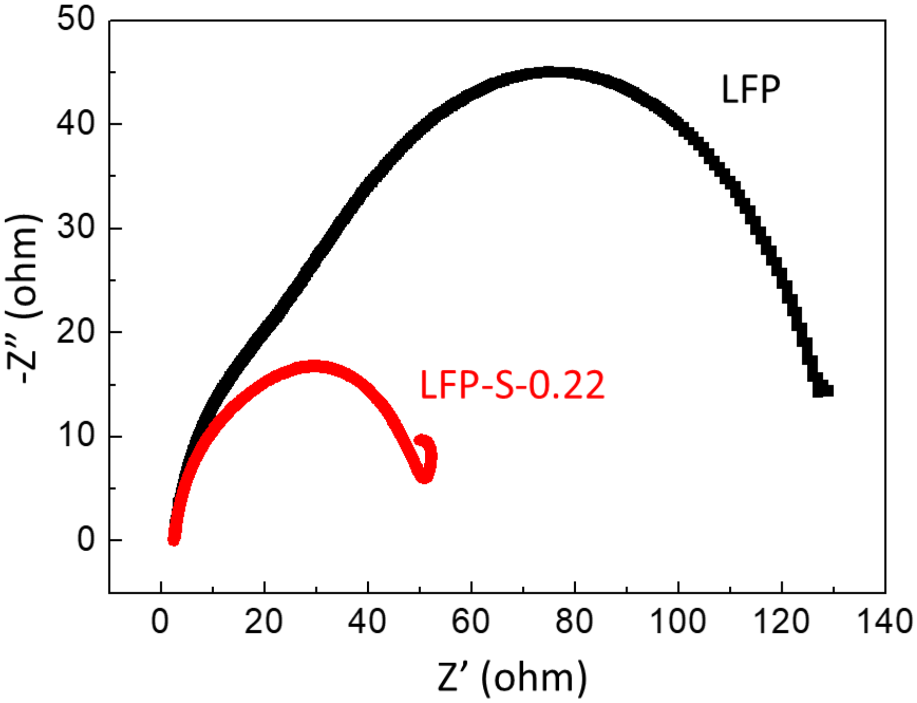

Figure S7. Electrochemical impedance spectra of LFP and LFP-S-0.22.

Table S2. Unit cell parameters and atom parameters for LEP-S-0.22 from Rietveld refinement (R_{wp} = 9.364, R_p = 7.101). There are three types of oxygen atoms in the olivine structure including two oxygen atoms occupied 4c sites (O1, O2), and one occupied 8d site (O3) as reported so far [for example, C. Gao, J. Zhou, G. Liu and L. Wang, *J. Alloys Compd.*, 2017, 727, 501]. The results of refinement with S-doping at the O1 site showed the smallest R_{wp} values compared to those at the O2 site, O3 site and randomly O sites, suggesting that S atoms is favored to be doped at the O1 site (the composition $LiFePO_{3.9}S_{0.1}$ estimated from EDS result was used for refinement).

Phase	Atom	Wyck.	x/a	y/b	z/c
	Li	4a	0.0	0.0	0.0
Pnma(62)	Fe	4c	0.28190(11)	0.25	0.97353(42)
	P	4c	0.09630(25)	0.25	0.42234(71)
a=10.30306(35) Å	O1	4c	0.09049(57)	0.25	0.74202(119)
b=5.99323(21) Å	O2	4c	0.45506(68)	0.25	0.21164(81)
c=4.7003(2) Å	O3	8d	0.16037(40)	0.05452(60)	0.28348(57)
